# Enteric Fever among Soldiers

**Published:** 1900-07-14

**Authors:** 


### ENTERIC FEVER AMONG SOLDIERS.

One of the most interesting of the addresses delivered before the recent meeting of the American Medical Association, held at Atlantic City, was that by Professsor Vaughan, of the University of Michigan, on the distribution of typhoid fever among the American soldiers in 1898. Its chief interest lay in the demonstration which it afforded of the practical certainty of typhoid fever arising within a couple of months or so of the assembly of an army in camp, and of the fact that the conditions of the camp and of camp discipline are the effective causes which determine whether or no the disease shall develop into an epidemic. He states that not only did every regiment in the service develop typhoid, but that more than 90 per cent. of the volunteer, and probably all of the regular regiments, developed the disease within eight weeks of assembling in the States camps. Another important point shown is that it did not require a very large aggregation of men to lead to this unfortunate result, for the disease became epidemic both in the small encampments of not more than one regiment as well as in the larger ones of one or more corps; in fact, so prevalent is typhoid fever in the States that it is more than probable that in any organisation of 1,300 men of military age, taken from private life and held together for two months, one or more cases will develop however perfect the sanitary condition of the camp may be. When, however, the disease has arisen all depends on the sanitary condition of the camps. The conclusion derived from these extended observations is that the whole question of the prevention of typhoid fever in armies is largely one of the disposal of excreta, and that the only way in which typhoid fever can with certainty be prevented is by the complete disinfection of the excreta of all, both the sick and the well. The practical recommendation is made that in place of the tubs, which are the worst form which camp conservancy can take, and the regulation pits which come next in the list, arrangements should be made in all permanent camps (where water carriage cannot be secured) for the excreta to be immediately disinfected and then carted away. For this purpose it is advised that galvanised iron troughs containing milk of lime should be used for the reception of all fecal matter, the contents being carted away daily beyond the precincts of the camp. This is a sensible and practical suggestion. Especial emphasis is laid upon the fact that the swarms of flies which surround both the tubs and the pits render futile all regulations as to covering up the excreta. The mischief is done before the cover can be placed, and direct observation showed that the flies swarm over the contents of the pits and then visit and feed on the food being prepared for the soldiers at the mess tents. It was even observed that, in some of the cases in which lime was used at the pits, flies with their feet whitened with lime were to be seen walking over the food. The presence of typhoid fever in an army so greatly takes away from its utility as a fighting force, that even from a merely military point of view no trouble would seem to be too great to take if by it such a scourge can be kept at bay, or even be diminished in its virulence. How great a scourge typhoid may become may be judged from the statement that about one-fifth of all the soldiers in the national encampments in the United States in 1898 developed typhoid fever.

